# Single-stage debridement via autogenous iliac bone graft through the OLIF corridor and lateral fixation in treating spontaneous single-level lumbar pyogenic spondylodiscitis

**DOI:** 10.1186/s12891-021-04815-3

**Published:** 2021-11-15

**Authors:** Songsong Wu, Bin Lin, Xi Li, Shengkai Chen, Haonan Zhang, Zhanglin Wu, Shenghui Tang, Yuehua Yang, Biru Liang

**Affiliations:** 1 Department of Orthopaedics, the Fifth Affiliated Hospital, Southern Medical University, No. 566 Congcheng Avenue, Conghua District, Guangzhou, 510900 People’s Republic of China; 2 Department of Orthopaedics, the Affiliated Southeast Hospital of Xiamen University, Zhangzhou, 363000 People’s Republic of China

**Keywords:** Lumbar spondylodiscitis, Oblique lateral interbody fusion, Minimally invasive

## Abstract

**Background:**

The aim of the present study was to investigate the efficacy and safety of mini-open oblique debridement and lumbar interbody fusion combined with lateral screw fixation for treating single-level pyogenic spondylodiscitis.

**Methods:**

Twelve patients with single-level lumbar pyogenic spondylodiscitis underwent OLIF combined with lateral screw fixation were analyzed. Patients underwent follow-up for 12 to 24 months. The clinical characteristics, etiological examinations, operative time, intraoperative blood loss, Oswestry Disability Index (ODI), visual analog scale score (VAS), postoperative complications, and the bony fusion rate were recorded.

**Results:**

The mean follow-up period of time was 14.8 months. The average operative time and intra-operative blood loss were 129.0 ± 19.76 min and 309.2 ± 92.96 mL, respectively. No severe intra-operative complications were observed during surgery, except in 1 case that develops abdominal pain and distension after surgery, 2 cases that develop left-sided transient thigh pain/numbness and 8 cases that complains of donor site (iliac crest) pain. All of these symptoms disappeared 8 weeks after surgery. Tissue sample cultures were obtained from all patients intraoperatively and four (33.3%) were positive, including 2 with Staphylococcus aureus, 1 with Staphylococcus epidermidis, and 1 with Escherichia coli. During an average of 22.5 ± 2.1 days (range, 14–29 days) after surgery, WBC, CPR, and ESR levels in all patients had returned to normal. All patients were pain free with no recurring infection. Solid bony fusions were observed in all cases within 6 months, including 10 with I grade fusion, 2 with II grade fusion according to the classification suggested by Burkus et al. No fixation failure was observed during follow up and solid bony fusions were observed in all 12 patients at finally follow-up. A significant postoperative increase was also observed in the mean segmental height and lordosis (*P* < 0.05), followed by a slight decrease of segmental height and lordosis at final follow-up. At the final follow up, the mean VAS (1.5 ± 0.6) and ODI (18.9 ± 7.6) were significantly lower than VAS (8.4 ± 2.7) and ODI (71.2 ± 16.5) before surgery (*P* < 0.01).

**Conclusion:**

Single-stage debridement with autogenous iliac bone graft through the OLIF corridor and lateral fixation was a feasible surgical approach in our consecutive 12 cases of pyogenic spondylitis.

## Introduction

The overall incidence of spondylodiscitis is approximately 2.2/100,000 per year [1, 2] and accounts for only 2–7% of all osteomyelitis [3]. Although Spondylodiscitis can affect patients of any age and most commonly occurs in adults (male vs female, 2:1). Especially, the high risk factors was susceptibility to spondylodiscitis including: aged>50 years, diabetes mellitus, intravenous drug abusers and chronic kidney or liver disease.

The most frequently involved spinal segment is the lumbar spine (58%), followed by the thoracic spine (30%), and the cervical spine (11%) [4]. Spondylodiscitis commonly results from primary hematogenous infection and is associated with destruction of the intervertebral disc, adjacent end-plates and vertebral body. Typically, pathogens of spondylodiscitis is caused by Staphylococcus species, Escherichia coli, and Mycobacterium tuberculosis. Typically, conservative treatment is used for most patients with pyogenic spondylodiscitis [5]. However, spondylodiscitis is difficult to treat because the positive rate of the causative organisms in spine is low. It is reported that 10–60% positive rate were obtained by fine-needle aspiration [5–7]. Ineffective conservative treatment may further worsen symptom and lead to serious complications, including epidural abscess, spinal kyphosis deformity, compression of nerves or neurological deficit symptoms [8, 9]. Therefore, surgery followed by treatment with antibiotics is required when conservative treatment fails [10]. It allows for effective debridement and rapid cure of inflammation.

However, the best surgical approach remains controversial. An anterior only approach debridement, a posterior only approach debridement, and a combined anterior–posterior debridement have been reported [11, 12]. However, the anterior lumbar interbody fusion (ALIF) has the potential risk for visceral and vascular injury, while posterior approach damages posterior structure and led instability of the spine [13–15]. Furthermore, the extreme lateral lumbar interbody fusion (XLIF) approach is associated with lumbar plexus injury risk [16, 17]. In addition, percutaneous endoscopic lavage and drainage is reported as an effective minimal invasive method for the treatment of the early-stage spinal infection patients. Obviously, this surgery is not suitable for patients with neurological deficit and mechanical instability [18–20].

Oblique lateral interbody fusion (OLIF), an approach accesses the spine between the abdominal anterior vessel and the psoas muscle [3], is considered the solution to the limitations of ALIF, XLIF and posterior lumbar interbody fusion (PLIF) [21]. Moreover, a previous research reported that OLIF in combination with posterior internal fixation was effective and safe for single-level spontaneous lumbar pyogenic spondylodiscitis [3]. However, it has never been reported as OLIF combined with lateral screw fixation for lumbar pyogenic spondylodiscitis.

In the present study, we retrospectively reviewed 12 cases of single-level spontaneous lumbar pyogenic spondylodiscitis that were treated in our hospital from December 2014 to December 2018 using the OLIF combined with lateral screw fixation. The aim of this study is to investigate the efficiency and safety of the OLIF combined with lateral screw fixation in treating pyogenic spondylodiscitis.

## Materials and methods

### Inclusion and exclusion criteria

Twelve consecutive patients (male 8 cases, female 4 cases) suffering from lumbar spondylodiscitis were enrolled. Lumbar pyogenic spondylodiscitis was confirmed in these patients on the basis of the following: back pain or leg pain accompanied by fever; Laboratory results including a culture study, erythrocyte sediment rate (ESR), white blood cells (WBC), and C-reactive protein (CRP), and the results of X-rays, CT scans, and MRI. These results were also confirmed by histopathological examination regardless of the bacteriological culture results [3]. The severity of spondylodiscitis was classified by Pee et al. [22] Grade I represents isolated discitis with minor destruction of vertebral endplates, Grade II represents discitis with moderate endplate destruction (including a portion of the vertebral body), and Grade III represents discitis with destruction of the vertebral body. All patients were included by the following inclusion and exclusion criteria.

**Inclusion criteria were as follows:**

(I) Diagnosis of single-segment pyogenic spondylodiscitis within the T12 to L5 areas;
(II) Persistent symptom and signs: infection, persistent pain, deterioration of neurological symptoms; progression, persistence, or recurrence of the disease failed to 6-week conservative treatment;
(III) Spontaneous pyogenic spondylodiscitis with no apparent cause;
(IV) Surgically treated using one-stage debridement, interbody fusion with autogenous iliac bone graft through the OLIF approach combined with lateral screw fixation.
(V) A minimal follow-up time beyond 12 months;

**Exclusion criteria were as follows:**

(i) Infection diagnosed as tuberculosis;
(ii) Secondary pyogenic spondylodiscitis;
(iii) Treatment with other approaches; or patients with posterior pathology requiring decompression and posterior fusion.

### Operative approach

#### Pre-surgery preparation

All patients were treated with conservative therapy including immobilization and administration of empirical broad-spectrum antibiotics for a minimum of 2 weeks. Each patient’s ESR, CRP, WBC levels were measured. All patients were evaluated with plain radiographs (X-rays), computed tomography (CT), and magnetic resonance imaging (MRI). The position of the psoas, anterior vasculature, posterior nerve structures and the kidneys was evaluated by CT and MRI.

#### Anesthesia and surgical position

All patients were continuously monitored during surgery using general anesthesia and each patient was positioned right lateral decubitus on a radiolucent table. The legs were only slightly flexed in order to prevent the patient from rolling on the bed. X-rays was used to confirm the target segment and mark the location for the initial incision.

#### Oblique approach and exposure

**Fig. 1:**
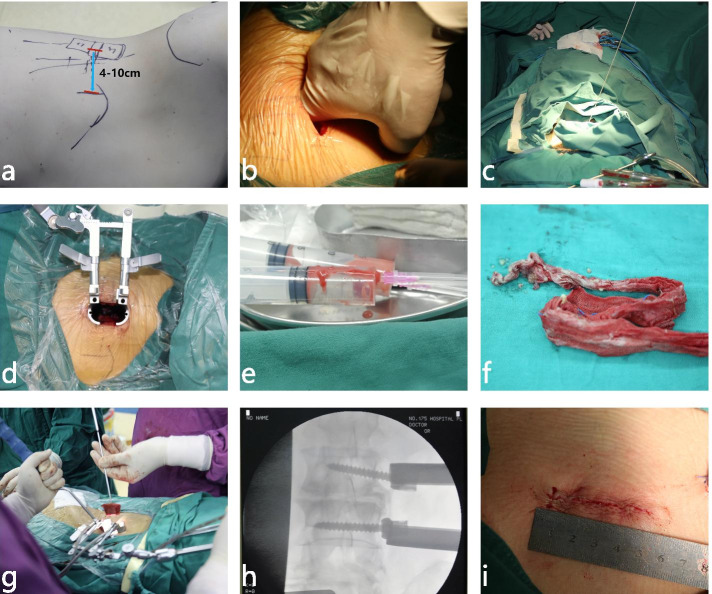
Steps of the OLIF combined with lateral fixation for lumbar spondylodiscitis. **a** Patient positioning and skin marking. **b** Blunt muscle dissection. **c** Placement of initial probe. **d** Dilation and retractor placement. **e** Bacterial culture of pyogenic fluids. **f** Clearing the focus completely with gauze. **g** Iliac bone grafting. **h** Lateral screw placement. **i** Wound closure

A 4–10 cm oblique incision parallel to the fiber of the external oblique abdominal muscle incision was made in the target spinal segment (Fig. 1a). Three muscular layers of the external oblique, the intra-abdominal oblique, and the transverse abdominis muscles were bluntly dissected and the retroperitoneal space was directly exposed by fingers of surgeon (Fig. 1b). Subsequently, a probe is guided down to the disc (Fig. 1c). The guidewire is placed through this probe and sequential dilation was performed to displace the surrounding tissues. A retractor is placed over the dilators (Fig. 1d).

#### Debridement and reconstruction

Then infectious lesions, sequestra, and all infected discs and endplate were meticulously debrided and cleared by syringe (Fig. 1e), followed by precise curettage to the healthy boundary (Fig. 1f). An appropriate autologous bone graft derived from the iliac crest was punched into the defect to reconstruct the anterior column after debridement, followed by lateral screw fixation was performed (Fig. 1g). The screws were placed on the adjacent vertebral body if the infected lesion exceeded 50% of the vertebrae and the screw was placed on the infected vertebral body if lesion less than 50%. X-ray examination was taken to confirm the appropriate internal fixation and the appropriate position of autologous bone graft (Fig. 1h). Finally, the drainage tube was put into the cavity and the incision was closed (Fig. 1i).

#### Post-surgery treatment

After the surgery, all patients were placed on strict bed rest for 7 days and ambulated with an assistive brace after 7 days. Tissue sample cultures were obtained from all patients intraoperatively and four (33.3%) were positive. Intravenous antibiotic therapy was continuously administered for a minimum of 6 weeks postoperatively based on the specific microbial sensitivities and the identified pathogenic organism. Oral antibiotics were not routinely used after discharge. A full course of broad-spectrum antibiotics was also administered for the patient with negative culture results.

#### Outcome measurements

The characteristics of patients, peri-operative laboratory, intra-operative blood loss and peri-operative complications were recorded. WBC, CRP, and ESR levels were assessed weekly after surgery until the results returned to normal. Clinical outcomes were evaluated using visual analog scale (VAS) score and oswestry disability index (ODI) score. The visual analogue scale (VAS) scoring system was used to evaluate pain level of patients from 0 (no pain) to 10 (very intense pain). The Oswestry disability index (ODI) is one of the most commonly used condition-specific outcome measures for spinal disorders. It includes 10 sections: walking, sitting, standing, pain intensity, sex life, social life, personal care, lifting, sleeping and traveling. All patients underwent X-rays, CT, and MRI of the lumbar spine before and after surgery. Specially, the bone graft healing, the segmental lordosis and height were assessed by X rays. X rays of the lumbar spine 1 day after surgery and again 1, 3, 6 and 12 months after surgery. The extent of bone graft healing was analyzed by the classification suggested by Burkus et al. [23] Grade I (definitely solid)—no motion on flexion-extension radiographs, continuous bony bridge, new bone formation adjacent on CT scan; Grade II (possibly solid)—no motion on dynamic radiographs, continuous bony incorporation, without evidence of new bone formation adjacent; Grade III (probably not solid)—no motion excluding evidence of bony incorporation; and Grade IV (definitely not solid)—motion on dynamic radiographs with no evidence of bony bridge. The absence of infection was defined as having no fever, pain, or graft bone union at the interface 12 months after surgery [24, 25].

#### Statistical analysis

All data were presented as mean ± standard error of mean (SEM). Datas were analyzed by Student t test using the SPSS19.0 program (SPSS Inc., Chicago, IL, USA) and a *p* value < 0.05 was considered statistically significant.

## Results

### Demographic data

A total of 12 cases (8 males and 4 female) were included according the inclusion and exclusion criteria. The average age was 56.5 years (range, 35–73 years), with a minimum follow-up duration of 12 months (mean, 14.8 mo; range 12–24 months). The mean duration of surgery was 129.0 ± 19.76 min. The mean blood volume loss during surgery was 309.2 ± 92.96 ml. During an average of 22.5 ± 2.1 days (range, 14–29 d) after surgery, WBC, CPR, and ESR levels in all patients had returned to normal. 3 cases complained of preoperative neurological defects preoperatively (Frankel Grade D) and all of them recovered to Frankel Grade E at 3-month follow-up. Tissue sample cultures were obtained from all patients intraoperatively and four (33.3%) were positive, including 2 with Staphylococcus aureus, 1 with Staphylococcus epidermidis, and 1 with Escherichia coli. A summary of the demographic data and surgical parameters are summarized in Table 1.

**Table 1:** Demographic data and surgical parameters of 12 patients

Case	Sex/Age	Disease level	Grade of infection	Surgery (min)	Blood Loss(mL)	Causative Organism	Frankel score (Pre/Post op)	Follow-up time (months)
1	M/49	L3–4	II	110	240	–	E/E	12
2	M/65	L2–3	III	96	230	Staphylococcus aureus	E/E	15
3	F/62	L3–4	II	125	300	–	D /E	18
4	M/59	L3–4	I	120	290	–	E/E	24
5	M/73	L4–5	III	135	250	Escherichia coli	E/E	12
6	M/41	L2–3	III	164	550	Staphylococcus epidermidis	D /E	15
7	F/55	L1–2	II	150	370	–	E/E	18
8	F/38	L3–4	II	148	310	Staphylococcus aureus	D/E	15
9	M/35	L2–3	III	137	420	–	E/E	12
10	M/71	L3–4	III	144	300	–	E/E	12
11	F/63	L3–4	I	106	210	–	E/E	12
12	M/67	L4–5	II	113	240	–	E/E	12
Average	56.5 ± 12.41	–	–	129.0 ± 19.76	309.2 ± 92.96	–	–	14.8 ± 3.56

### Clinical outcome

All patients were back and radicular pain free at finally follow-up. The mean preoperative, 1-month postoperative, and final follow-up VAS scores were 8.4 ± 2.7, 2.0 ± 0.5 and 1.5 ± 0.6 respectively. The mean preoperative, 1-month postoperative, and final follow-up ODI scores were 71.2 ± 16.5, 32.5 ± 8.1, and 18.9 ± 7.6 respectively. These follow-up VAS and ODI scores showed statistical significances compared with preoperative values (independent t test, *P* < 0.01) (Table 2).

**Table 2:** VAS scores and ODI scores

Item	Pre-op	1mo Post-op	3mo Post-op	Final follow-up
VAS	8.4 ± 2.7	2.0 ± 0.5	1.8 ± 0.5	1.5 ± 0.6
ODI	71.2 ± 16.5	32.5 ± 8.1	21.2 ± 8.4	18.9 ± 7.6

### Imaging measurements

**Fig. 2:**
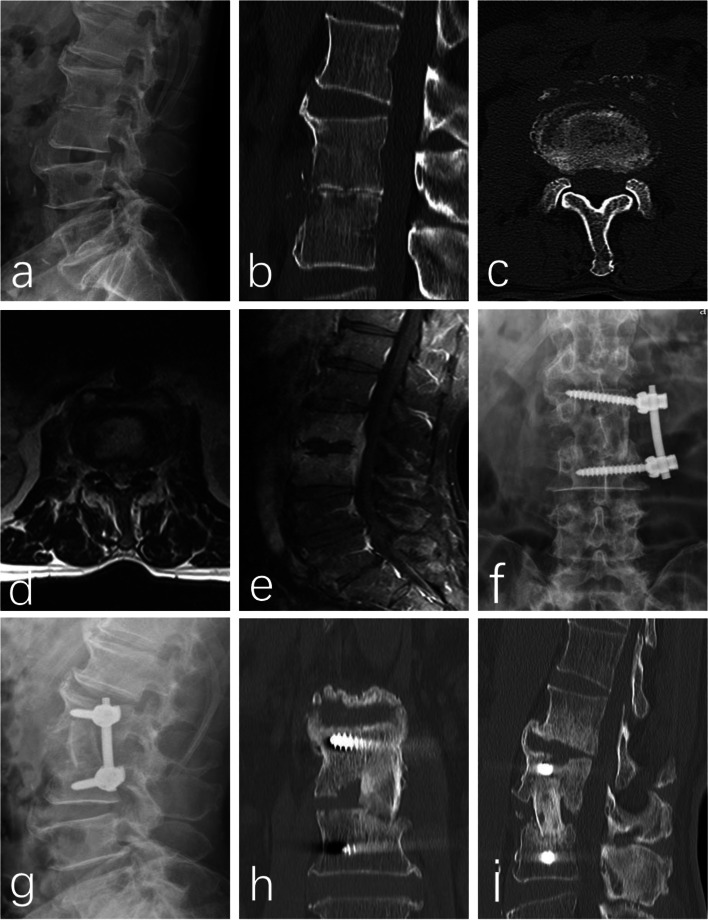
Sixty-five-year-old male with lumbar spondylodiscitis of L_2–3_. **a** Pre-op X-ray demonstrating disc space narrowing at L_2–3_. **b, c** Pre-op CT demonstrating bone destruction. **d, e** Pre-op MRI demonstrating abnormal signal, soft tissue swelling, and spinal cord compressed. **f, g** CT at 3 days after operation demonstrating Segmental lordosis and height was restored. **h, i** CT at 6 months follow-up demonstrating bony fusion

**Fig. 3:**
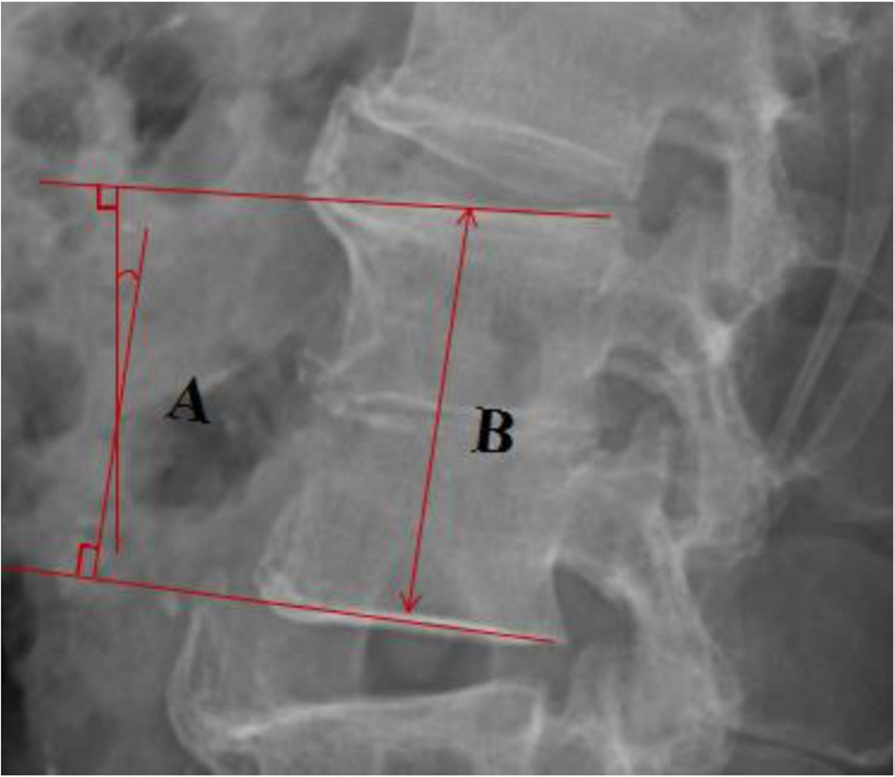
Measurement of segmental lordosis (**A**) and height (**B**). **A**: The lordotic angle was measured by the angle of perpendicular lines from the upper and lower endplates. **B**: The segmental height was measured as the midpoint distance from the endplates

No internal fixation failure was observed in the X-ray or CT during follow up (a case is shown in Fig. 2). According to the classification suggested by Burkus et al [23], 10 cases showed grade I fusion status, which was assessed with CT and flexion-extension radiographs at 6 months postoperatively follow-up. 2 case showed grade II fusion status. Neither of them complained of any clinical symptoms related to pseudoarthrosis and solid bony fusions were observed in all 12 patients at 12 months postoperatively follow-up. As shown in Table 3, a significantly postoperative increase was also observed in the mean segmental height and lordosis (*P* < 0.05), followed by a slight decrease of segmental height (*P* < 0.05) and lordosis at final follow-up (Measurement is shown in Fig. 3). However, there was also a significant difference in segmental lordosis and height at final follow-up, as compared with that in preoperative group. The data suggested that restoration of segmental lordosis and height were successfully achieved by the surgery and maintained at final follow-up.

**Table 3:** Changes in segmental lordosis and height

Item	Pre-op	Post-op	Final follow-up
segmental lordosis	1.5 ± 4.1°	8.5 ± 5.6°	5.3 ± 4.7°
segmental height	62.3 ± 9.3 mm	70.6 ± 10.4 mm	66.5 ± 9.7 mm

### Complication

No incision infection, ureter injury, retrograde ejaculation, spinal nerve injury, major vessel injury, urinary injury or hardware failure were observed during surgery. only 1 (8.3%) case developed abdominal pain and distension after surgery, which was effectively relieved after fasting and laxative measures for 3 days. 2 cases (16.6%) developed left-sided transient thigh pain/numbness and the symptom diminishes after 2 weeks of surgery. 8 cases complained of donor site (iliac crest) pain but all of them relieve after 8 weeks of surgery. In addition, no infections had recurred.

## Discussion

For most patients with pyogenic spondylodiscitis, conservative therapy is an effective economical and safe treatment [5, 26, 27], but surgical intervention is required when it develops epidural abscess, spinal kyphosis deformity, compression of nerves or neurological deficit symptoms [8, 9]. The purpose of surgical treatment for spondylodiscitis is to debride the lesion, decompress the nerve, relieve the symptoms, correct the spinal deformity, and reconstruct spinal stability. In this study, causative organisms were identified in 4 patients (33.3%)*,* which was comparable to previous reports that the pathogen could be detected in 30 to 83% of cases [27]. The implantation of osteosynthesis material in an infected wound area is controversy because it may increase the risk of metal surface microbial colonization and lead to persistent infection. However, recent studies demonstrated that bone grafting followed internal fixation could improve spine stability, promote bone fusion, but did not increased rate of infection [10, 12, 27, 28]. Therefore, debridement following bone grafting and internal fixation is the current standard surgical protocol for treatment of spondylodiscitis. However, controversy remains over whether internal fixation should be done in a single stage or in two stages after debridement, the choice of surgical approach and internal fixation.

Spinal infection is usually involved in vertebral bodies and discs, so anterior approach is often recommended to debride the lesion, reconstruct spinal stability and achieve better clinical results [12]. Several researchers reported that ALIF or XLIF corridor followed by a posterior stabilization procedure is also an option for spondylodiscitis [11, 14]. However, these ALIF or XLIF debriding approaches have certain risks, including major vascular injuries, the psoas major muscle and lumbar plexus injuries, urinary retention, constipation, and pain/numbness in the thigh [11, 14, 16].

OLIF approach was first reported by Mayer and applied to lumbar disc disease through the natural space between the lateral border of the abdominal vessel and the psoas muscle [29]. In this study, no severe nerve or vascular injuries were observed during and after surgery, which suggested that the OLIF was a safe approach. only 1 case developed abdominal pain and distension after surgery, which was effectively relieved after fasting and laxative measures for 3 days. 2 cases (16.6%) developed left-sided transient thigh pain/numbness and the symptom diminished 2 weeks after surgery. Consistently, it is reported that the incidence of transient thigh pain/numbness occurred in 8.3–20.4% and permanent thigh pain/numbness occurred in 4–5% in these cases treated with OLIF [30–33]. Thus, the bone grafts in all patients were fused within 12 months after surgery, which was comparable to the results presented in previous reports [34, 35].

Segmental lordosis and height were restored satisfactory at the immediate postoperative period in all 12 cases, because the OLIF approach allows for wider exposure of the disc space and the size of the debridement area can be directly measured to fill in the defect and better stabilize the anterior column. It maintains the segmental lordosis and height although a slight decrease were observed at final follow-up, suggesting that it is an effective anterior grafting with autologous bone strut. In our study, 8 cases complained of donor site (iliac crest) pain. To reduce this complication, researchers suggest that cages instead of autologous bone may be a viable option for single-stage anterior reconstruction [22, 36, 37]. However, it is notable that the high incidence cage subsidence (range from 10 to 13.6%) were reported [28, 37, 38]. Furthermore, cages instead of autologous bone may lead to a higher medical burden for these patients. In addition, VAS and ODI scores at 12 months after surgery had significantly improved over those before surgery, which is comparable to the results presented in a previous report treated with OLIF combined with posterior pedicle screws [3]. However, OLIF corridor combined with lateral screw theoretically avoid additional iatrogenic injury to the posterior structures.

## Conclusion

Single-stage debridement with autogenous iliac bone graft through the OLIF corridor and lateral fixation is an effective and safe surgical approach in our consecutive 12 cases of pyogenic spondylitis.. However, further studies with longer follow-up and more patients are still needed.

## Data Availability

The data that support the findings of this study are available from the corresponding authors but restrictions apply to the availability of these data, which were used under license for the current study, and so are not publicly avail-able. Data are however available from the authors upon reasonable request and with permission of the hospital ethical institutional review board.

## Abbreviations

OLIF: Oblique lateral interbody fusion
ODI: Oswestry Disability Index
VAS: Visual analog scale score
WBC: White blood cell
CPR: C-reactive protein
ESR: Erythrocyte sedimentation rate
ALIF: Anterior lumbar interbody fusion
XLIF: Extreme lateral lumbar interbody fusion
PLIF: Posterior lumbar interbody fusion
CT: Computed tomography
MRI: Magnetic resonance imaging
SEM: Standard error of mean
